# Antifungal Therapy: New Advances in the Understanding and Treatment of Mycosis

**DOI:** 10.3389/fmicb.2017.00036

**Published:** 2017-01-23

**Authors:** Liliana Scorzoni, Ana C. A. de Paula e Silva, Caroline M. Marcos, Patrícia A. Assato, Wanessa C. M. A. de Melo, Haroldo C. de Oliveira, Caroline B. Costa-Orlandi, Maria J. S. Mendes-Giannini, Ana M. Fusco-Almeida

**Affiliations:** Laboratório de Micologia Clínica, Departamento de Análises Clínicas, Universidade Estadual Paulista (UNESP), Faculdade de Ciências FarmacêuticasAraraquara, Brasil

**Keywords:** antifungal drugs, antifungal resistance, biofilms, fungal vaccine, new antifungal therapy, alternative animal models, nanoparticles

## Abstract

The high rates of morbidity and mortality caused by fungal infections are associated with the current limited antifungal arsenal and the high toxicity of the compounds. Additionally, identifying novel drug targets is challenging because there are many similarities between fungal and human cells. The most common antifungal targets include fungal RNA synthesis and cell wall and membrane components, though new antifungal targets are being investigated. Nonetheless, fungi have developed resistance mechanisms, such as overexpression of eﬄux pump proteins and biofilm formation, emphasizing the importance of understanding these mechanisms. To address these problems, different approaches to preventing and treating fungal diseases are described in this review, with a focus on the resistance mechanisms of fungi, with the goal of developing efficient strategies to overcoming and preventing resistance as well as new advances in antifungal therapy. Due to the limited antifungal arsenal, researchers have sought to improve treatment via different approaches, and the synergistic effect obtained by the combination of antifungals contributes to reducing toxicity and could be an alternative for treatment. Another important issue is the development of new formulations for antifungal agents, and interest in nanoparticles as new types of carriers of antifungal drugs has increased. In addition, modifications to the chemical structures of traditional antifungals have improved their activity and pharmacokinetic parameters. Moreover, a different approach to preventing and treating fungal diseases is immunotherapy, which involves different mechanisms, such as vaccines, activation of the immune response and inducing the production of host antimicrobial molecules. Finally, the use of a mini-host has been encouraging for *in vivo* testing because these animal models demonstrate a good correlation with the mammalian model; they also increase the speediness of as well as facilitate the preliminary testing of new antifungal agents. In general, many years are required from discovery of a new antifungal to clinical use. However, the development of new antifungal strategies will reduce the therapeutic time and/or increase the quality of life of patients.

## Introduction

When compared with antibacterial research, little progress has been made in the development of new antifungal agents, which has been justified by the low occurrence of fungal infections. However, the current increase in incidence of fungal infections has led to aggressive research on new antifungal agents as evidenced by the rise in the number of publications since the 1960s (Maertens, 2004; Ngo et al., 2016). Another reason for the slow development of antifungal agents is the fact that fungi are eukaryotic, with a close evolutionary relationship with human hosts, which complicates the search for antifungal targets. Nonetheless, detailed knowledge regarding the structure, composition and biochemistry of fungal cells, in addition to various facets of fungal infections, has contributed to our understanding about the mechanism of action of many antifungal agents (Borgers, 1980; Kanafani and Perfect, 2008). Typically a long period of 8 to 10 years is required for an antifungal to be approved for clinical use. Reducing toxicity, enhancing bioavailability, improving the antifungal spectrum and combating resistance are efforts that are expected to increase the efficacy of the available antifungals. Indeed, elucidation of the mode of action of a potential antifungal compound can shorten the time from lead to candidate drug. Small antifungal molecules from natural products could represent structural templates for structure-activity relationship studies, thus providing more information to optimize potential new antifungal agents (Sheng and Zhang, 2011). Overall, new strategies regarding antifungal therapy, target identification and rational drug design technologies can significantly accelerate the process of new antifungal development, reducing the time to cure or providing better quality of life to patients.

## Antifungal Mechanism of Action: Old and New Targets of Antifungal Candidates

**FIGURE 1:**
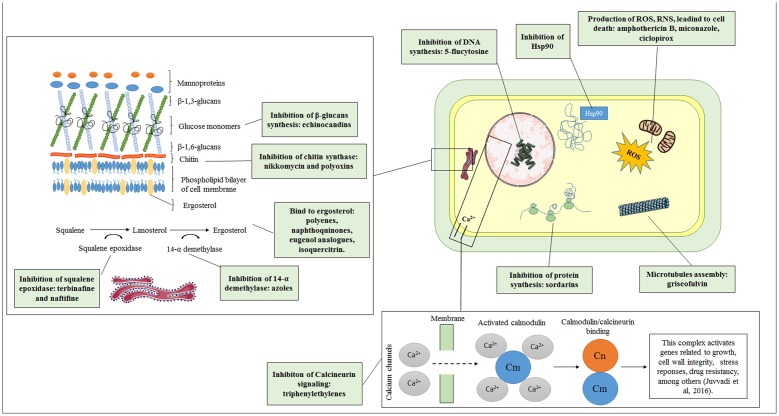
**Old and new targets as antifungal candidates**.

Although the commercially available antifungal agents to date have targets that are restricted to the plasma membrane and the cell wall (Odds et al., 2003; Sundriyal et al., 2006; Ngo et al., 2016), a certain diversity of targets has been discovered. To develop new therapies, recent studies have focused on the inhibition of fungal virulence factors. Some mechanisms of action are described below, and an overview is presented in **Figure 1**.

### Ergosterol

Ergosterol is a lipid responsible for membrane fluidity and permeability and for the function of fungal integral membrane proteins; accordingly, this sterol is essential for cell viability (Leber et al., 2003; Tatsumi et al., 2013; Song et al., 2016). Several antifungals primarily target ergosterol, either by inhibiting its biosynthesis or by binding to it, causing formation of pores in the membrane.

Azole antifungals act by inhibiting ergosterol biosynthesis via the cytochrome P450 enzyme 14-α demethylase, which catalyzes the conversion of lanosterol to ergosterol (Kathiravan et al., 2012). Azoles affect the integrity of fungal membranes, altering their morphology and inhibiting growth (Kathiravan et al., 2012; Tatsumi et al., 2013).

The allylamine class, represented by terbinafine and naftifine, function by inhibiting the early steps of fungal ergosterol biosynthesis (Kathiravan et al., 2012) by targeting the enzyme squalene epoxidase, encoded by ERG1. This inhibition leads to accumulation of squalene and the absence of other sterol derivatives. Allylamines are highly effective against dermatophytes because they have been shown to accumulate more in the skin and nail beds relative to the blood, possibly due to their lipophilicity (Ngo et al., 2016).

Polyenes, such as nystatin and amphotericin B, exhibit fungicidal activity primarily by binding to ergosterol to form a complex capable of disrupting the membrane and leading to leakage of monovalent ions as well as other cytoplasmic contents (Odds et al., 2003; Walsh et al., 2008; Baginski and Czub, 2009). A second mechanism of polyene action involves a cascade of oxidation reactions and interactions with lipoproteins that impair membrane permeability through the release of free radicals (Sangalli-Leite et al., 2011; Mesa-Arango et al., 2012, 2014).

### Cell Wall

The fungal cell wall, which is primarily composed of chitin, glucans, mannans, and glycoproteins, is essential for adhesion and fungal pathogenesis and also serves as a protective barrier, limiting the access of molecules to the plasma membrane (Bowman and Free, 2006; van der Weerden et al., 2013). The two main mechanisms of action of antifungals targeting the cell wall are related to the inhibition of chitin and β-glucan synthesis.

In the period between 2001 and 2006, the echinocandin class of drugs, represented by caspofungin, micafungin, and anidulafungin, was developed. This class has different mechanisms of action that are specific for the fungal cell wall. Echinocandins target the protein complex responsible for the synthesis of β-1,3 glucans by blocking the enzyme glucan synthase (Odds et al., 2003). This blockage causes a decrease in the incorporation of glucose monomers linking β-1,3 and β-1,6 glucans, thereby weakening the cell wall and leading to fungal cell lysis (Kathiravan et al., 2012; Song and Stevens, 2016).

Chitin, a β-1-4-linked *N*-acetylglucosamine polymer, is an essential component of the fungal cell wall, though it is only present in very small amounts in yeasts (1–2%) but in considerable quantities in filamentous fungi (10–20%) (Bowman and Free, 2006; Morozov and Likhoshway, 2016). Nikkomycin and polyoxins are antifungal agents that target chitin synthase, which is responsible for elongation of the chitin chain and, therefore, is considered an attractive target (Kathiravan et al., 2012).

### Inhibition of Nucleic Acid, Protein, and Microtubule Syntheses

Inhibition of nucleic acid synthesis is related to the action of 5-flucytosine, which is converted primarily to 5-fluorouracil by the enzyme cytosine deaminase and then to 5-fluorouridylic acid by UMP pyrophosphorylase (Odds et al., 2003). Although 5-flucytosine was synthesized in 1957, its antifungal property was not discovered until 1964, 7 years later (Shukla et al., 2016). This acid can be incorporated into RNA, resulting in premature chain termination, thereby inhibiting DNA synthesis through effects on the enzyme thymidylate synthase (Polak and Scholer, 1975; Odds et al., 2003; Kathiravan et al., 2012).

With respect to the synthesis of microtubules, it is known that griseofulvin interferes with intracellular production, thus inhibiting fungal mitosis (Kathiravan et al., 2012).

Finally, sordarins suppress protein synthesis, which retards cell growth. Essentially, two fungal proteins have been described as target of sordarins: translation elongation factor 2 (eEF2) and the large ribosomal subunit protein rpP0 (Botet et al., 2008). Attempting to elucidate the mechanism of action, Justice et al. (1998) performed genetic assays using *Saccharomyces cerevisiae* mutants to demonstrate the fungal specificity of sordarins and proved that eEF2 is a target.

### Reactive Oxygen Species (ROS)

It is known that treatment with some antifungals such as AmB and itraconazole can cause more than one effect on fungal cells (Ferreira et al., 2013; Mesa-Arango et al., 2014). According to Mesa-Arango et al. (2014), mitochondria naturally produce free radicals. However, under adverse conditions, such as in the presence of oxidants and UV light, these free radicals are produced in abundance, causing damage to proteins, lipids and DNA and leading to cell death. Accordingly, ROS production is also associated with apoptosis. Treatment with AmB is able to induce oxidative and nitrosative bursts in *Candida*, *Cryptococcus*, and *Trichosporon*, enhancing its fungicidal effect (Ferreira et al., 2013; Mesa-Arango et al., 2014).

### Inhibition of Heat Shock Protein 90 (Hsp90)

Heat shock protein 90 (Hsp90) is a molecular chaperone of the heat shock protein (Hsp) family. Synthesized as an adaptive response to noxious conditions, these proteins contribute to the survival of pathogenic microorganisms in the host (Jacob et al., 2015). Hsp90 has been related to fungal pathogenicity, phase transition in dimorphic fungi and antifungal drug resistance, making it a potential target for antifungal therapy (Burnie et al., 2006; Brown et al., 2010; Jacob et al., 2015). Jacob et al. (2015) examined the transcription profiles of *Trichophyton rubrum* under different stress conditions, such as interaction with nail and skin cells and molecules, nutrients and treatment with antifungal drugs. In addition to suggesting the role of Hsp90 in the pathogenesis and susceptibility to dermatophytosis antifungal agents, the authors also related this protein to the regulation of other heat shock proteins.

### Inhibition of Calcineurin Signaling

Calcineurin is defined as a conserved Ca^2+^-calmodulin(CaM)-activated protein phosphatase 2B that belongs to the phosphor-protein phosphatase family (Juvvadi et al., 2016). This protein is involved in calcium-dependent signaling and regulation of several important cellular processes in yeasts (*Candida* spp., *Cryptococcus* spp.) and filamentous fungi (*Aspergillus fumigatus*), including growth, cell wall integrity, transition between morphological states, cation homeostasis, stress responses, and drug resistance (Blankenship et al., 2003; Steinbach et al., 2006; Chen et al., 2013; Juvvadi et al., 2016). More important is the role of calcineurin in maintaining the integrity of the fungal cell wall by regulating downstream effectors and influencing the biosynthesis of ergosterol, chitin and β-glucans. Odom (2014) suggested that triphenylethylenes, described by Butts et al. (2014), are a novel class of antifungal drugs that act on calcium homeostasis in *Cryptococcus neoformans* via direct inhibition of the calcineurin activator calmodulin.

## Molecular Mechanisms of Antifungal Resistance

**FIGURE 2:**
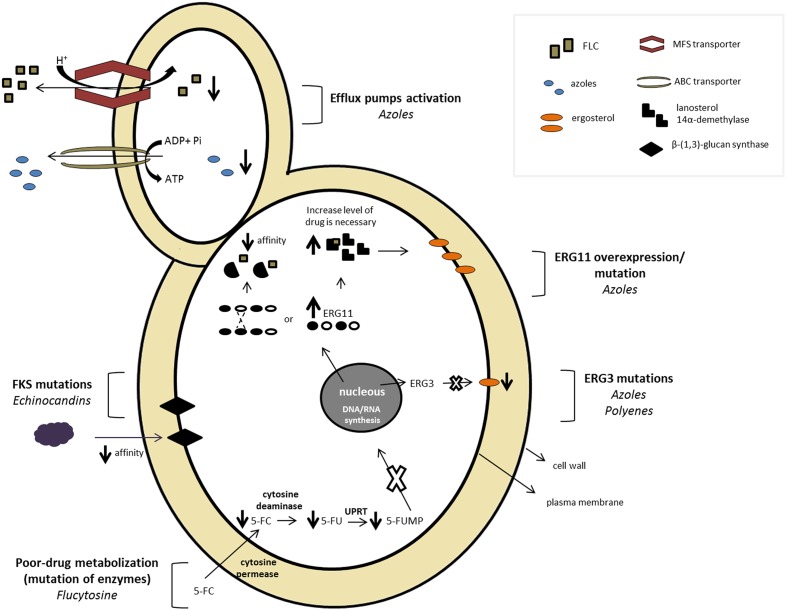
***Candida* sp. mechanisms of resistance to different antifungal classes**.

The widespread use of antifungal agents and the limited arsenal associated with the increased number of opportunistic infections have resulted in the progression of resistance to available drugs. The antifungal resistance mechanism may occur through different conditions such as a decrease in the effective drug concentration, changes or overexpression of the drug targets, and metabolic bypasses (Sanglard, 2016). **Figure 2** depicts an overview of several antifungal resistance mechanisms described for *Candida* spp.

### Molecular Mechanisms of Resistance to Azoles

Azole resistance includes the following mechanisms: (1) activation of eﬄux pumps, (2) qualitative changes in the target enzyme, (3) quantitative changes caused by overexpression of ERG11, and (4) alterations in cell wall composition.

#### Activation of Eﬄux Pumps

Reduction in intracellular antifungal accumulation in *Candida* spp. is a consequence of the overexpression of membrane-associated transporters acting as multidrug eﬄux pumps (Prasad and Rawal, 2014). Two main classes of transporters are described as being involved in this resistance mechanism. The superfamily of ATP-binding cassette (ABC) proteins comprises the primary activity, hydrolyzing ATP to provide energy to drive the eﬄux of drugs. Transporters belonging to the major facilitator superfamily (MFS) constitute the secondary activity; these pumps utilize a proton electrochemical gradient across the plasma membrane to extrude substrates (Cannon et al., 2009). Although most *Candida* species are naturally susceptible to azoles, an increasing number of cases of acquired resistance have been reported in clinical isolates of patients exposed to prolonged treatment, especially to FLZ (White et al., 1998; Pfaller and Diekema, 2007; Arendrup, 2014; Espinel-Ingroff et al., 2014).

*Candida albicans* azole-resistant isolates can overexpress one or more eﬄux pumps (White, 1997; Franz et al., 1998; Lopez-Ribot et al., 1998). *C. albicans* possesses 28 putative types of ABC transporters, two of which, CDR1 and CDR2, are well characterized and overexpressed in resistant-isolates. Cdr1 has a greater contribution to FLZ resistance than Cdr2 (Sanglard et al., 1997; Sanglard et al., 2009; Prasad and Goffeau, 2012). Additionally, gain-of-function mutation of TAC1 (transcriptional activator of CDR genes) is related to increased levels of CDR1 and CRD2 (Coste et al., 2007). Regarding MFS transporters, *C. albicans* has 96 potential MFS transporters, though only one has been described with respect to azole resistance, the MDR1 member of the DHA1 family (Gaur et al., 2008).

It has been postulated that CDR genes in *C. albicans* are involved in the removal of different azoles. CDR1 eﬄux is associated with a wide range of substrates, whereas MDR1 appears to be specific for FLZ, as its overexpression results in moderate resistance to FLZ (Sanglard et al., 1995; White et al., 1998; Hiller et al., 2006; Cheng et al., 2007). The ABC and MFS pumps differ in relation to their structures and mechanisms, and differences in substrate specificity are expected (Keniya et al., 2015).

The exact mechanism underlying FLZ resistance in *C. glabrata* is not well defined; however, different studies indicate an essential role for ABC transporters (Abbes et al., 2013). FLZ-resistant *C. glabrata* clinical isolates present CDR1 and CDR2 upregulation (Sanglard et al., 1999; Bennett et al., 2004). Sanguinetti et al. (2005) observed increased expression of SQN2 (another ABC transporter) in two isolates (among 29) expressing normal levels of CRD1 and CRD2; SQN2 is possibly involved in the resistance of this species.

Studies have demonstrated that the primary mechanisms of azole resistance in clinical isolates of *C. dubliniensis* is upregulation of Mdr1 (Moran et al., 1998). Mdr1 is invariably overexpressed in *C. dubliniensis* strains with reduced susceptibility to FLZ (Perea et al., 2001). Moreover, among clinical isolates obtained from HIV-infected individuals with oropharyngeal candidiasis, Perea et al. (2002) reported upregulation of Mdr1 in all isolates with a high FLZ-resistance level, whereas less than half of the isolates presented CDR upregulation. Despite the 92% identity of the *C. dubliniensis* CDR1 gene with *C. albicans*, this may occur because *C. dubliniensis* genotype 1, previously described by Gee et al. (2002), possesses a non-sense mutation in CDR1 that converts a normal codon to a stop codon (TAG), resulting in expression of a truncated protein of 85 kDa instead of the wild-type 170 kDa (Perea et al., 2002).

*Candida krusei* carries two homologous genes for ABC transporters previously described for *C. albicans*: ABC1 and ABC2. The ABC1 gene is upregulated in cultures exposed to imidazole and cycloheximide. However, cycloheximide exhibits antagonist activity against FLZ, and this correlation between antagonism and upregulation suggests that FLZ and other azoles may be substrates for the ABC1 transporter (Katiyar and Edlind, 2001). Thereafter, Lamping et al. (2009) showed the involvement of ABC1 in the innate azole resistance of *C. krusei*, and Guinea et al. (2006) found that MDR proteins have a role in *C. krusei* resistance. However, expression of MFS transporters was not reported determined in FLZ-resistant *C. krusei*.

The FLZ resistance of *C. parapsilosis* has been linked to overexpression of MRR1 in high association with a mutation (Zhang et al., 2015) that concomitantly results in overexpression of MDR1 and overexpression of CRD1 (Souza et al., 2015; Zhang et al., 2015). The involvement of eﬄux pumps in *C. tropicalis* clinical isolate antifungal resistance has not yet been observed, but there is an *in vitro* study demonstrating the development of FLZ-resistance associated with the up-regulation of MDR1 and CDR1 (Barchiesi et al., 2000).

The involvement of eﬄux pumps has also been described in other pathogenic fungi. In *C. neoformans*, overexpression of the AFR1 gene, of the ABC transporter family, is associated with FLZ resistance (Posteraro et al., 2003; Sanguinetti et al., 2006). In addition, afr2p and Mdr1p, members of the ABC and MFS families, respectively, from *C. neoformans* and *C. gattii* can also promote resistance to FLZ and other azoles (Basso et al., 2015).

The ABC transporter Afr1 also acts in azole resistance in *Aspergillus* because overexpression of the AFR1 gene from *A. fumigatus* can confer itraconazole resistance (Slaven et al., 2002). Although *Aspergillus fumigatus* has four Mdr-like eﬄux pumps, itraconazole-resistant isolates or strains under itraconazole treatment only present overexpression of *mdr3* and *mdr4* (da Silva Ferreira et al., 2004).

Eﬄux appears to be the most prevalent mechanism of resistance in dermatophytes. Increased expression of the ABC transporters genes TruMDR1 and TruMDR2 from *T. rubrum* is observed in the presence of azoles (Cervelatti et al., 2006; Fachin et al., 2006). The use of eﬄux pumps by dermatophytes is also implicated in resistance to other antifungals, such as terbinafine, amphotericin B and griseofulvin (Cervelatti et al., 2006; Paião et al., 2007; Yu et al., 2007; Ghannoum, 2016; Martins et al., 2016).

#### ERG11: Overexpression and Changes in Drug Targets

In the presence of FLZ, *C. albicans* increases ERG11 expression, most likely via compensatory mechanisms, to deplete ergosterol (Albertson et al., 1996). However, Franz et al. (1998) showed that even in the absence of FLZ, resistant isolates express ERG11 at higher levels compared to susceptible isolates exposed to the drug. Overexpression of ERG11 results in increased concentrations of lanosterol 14α-demethylase; consequently, larger amounts of the antifungal are required to inhibit the enzyme (Morschhäuser, 2002). This mechanism has been described for many *C. albicans* FLZ-resistant isolates (White et al., 2002; Chau et al., 2004; Goldman et al., 2004). ERG11 overexpression in azole-resistant isolates can occur via two mechanisms of ERG11 amplification. One is formation of an isochromosome containing two copies of the left arm of chromosome 5, where ERG11 resides, and duplication of the entire chromosome (Selmecki et al., 2006). The second mechanism is mutation in the zinc cluster finger transcription factor Upc2, which results in overexpression of ERG11 in *C. albicans* (Dunkel et al., 2008). Overexpression of ERG11 has also been reported for other azole-resistant *Candida* species; however, the mechanisms remain unknown (Barchiesi et al., 2000; Redding et al., 2002; Vandeputte et al., 2005; Rogers, 2006; Jiang et al., 2013; Cowen et al., 2014).

Another mechanism of azole resistance among *Candida* strains involves non-synonymous point mutations in the ERG11 gene, which encodes the target enzyme lanosterol 14α-demethylase. Mutations in ERG11 can result in post-translational modifications in the amino acid sequence and consequently in the three dimensional structure of Erg11p, causing decreased binding affinity for azole components and also reducing ergosterol biosynthesis without impeding enzyme function but generating yeast with an altered phenotype resistant to azole (Xiang et al., 2013). Among 160 different amino acid substitutions, only 10 have been confirmed in FLZ resistance; four were obtained in the laboratory but not yet detected in clinical isolates (Chen et al., 2007). The substitutions R467K, I471T, G464S, S405F, and K143R were only related to azole-resistant *C. albicans* isolates (Sanglard et al., 1998; Lamb et al., 2000; Morio et al., 2010), with the most common being R467K and G464S (Morio et al., 2010). ERG11 mutations have been described for other azole-resistant clinical *Candida* isolates, including *C. dubliniensis* (Perea et al., 2002), *C. krusei* (Ricardo et al., 2014), *C. tropicalis* (Vandeputte et al., 2005; Jiang et al., 2013) and more recently *C. parapsilosis* (Grossman et al., 2015). However, the same finding has not yet described for *C. glabrata* (Gonçalves et al., 2016).

In non-*Candida* species, the most common mechanism of azole resistance is alteration of the target protein. Several studies describe point mutations in the ERG11 (CYP51) gene, encoding 14-α-demethylase, leading to amino acid substitution that decreases the affinity for azole (Xie et al., 2014).

In *Cryptococcus* spp., three point mutations in ERG11 have been described in association with azole resistance: point mutation G1855T leading to amino acid substitution of glycine 484 with a serine (G484S) (Rodero et al., 2003); substitution of tyrosine 132 by phenylalanine occurring in the catalytic domain (Sionov et al., 2012); and point mutation G1855A, also resulting in the amino acid substitution G484S (Bosco-Borgeat et al., 2016). *Cryptococcus neoformans* under FLZ stress is also able to adapt via duplication of chromosome 1, on which the genes ERG11 and AFR1 and that encoding the FLZ transporter protein are found (Sionov et al., 2010).

*Aspergillus* spp. have two genes encoding 14-α-demethylase: CYP51A and CYP51B. However, azole resistance is more associated with mutation in *CYP51A* (Gonçalves et al., 2016). Different point mutations and non-synonymous mutations have been described in *CYP51A*, and the different patterns of azole resistance depend on these mutations (Diaz-Guerra et al., 2003; Mann et al., 2003; Chowdhary et al., 2014a; Leonardelli et al., 2016). For example, non-synonymous mutations in codons 98,138, 220, 431, 434, and 448 may confer resistance to all azoles in *Aspergillus* spp. (Howard et al., 2009).

In drug-resistant *Aspergillus*, tandem repeat mutation associated with amino acid substitution in the *CYP51A* promoter have been described, such as TR_34_/L98H and TR46/Y121F/T289A (Verweij et al., 2007; Vermeulen et al., 2013). The most prevalent is TR_34_/L98H, which is found in different resistant *Aspergillus* isolates worldwide and seems to be the major mutation associated with azole resistance (Lockhart et al., 2011; Chowdhary et al., 2012, 2014b; Badali et al., 2013; Seyedmousavi et al., 2013; Chen et al., 2016).

In *Histoplasma capsulatum*, a Y136F substitution in CYP51Ap has also been implicated in decreased susceptibility to FLZ and voriconazole (Wheat et al., 2006).

#### Erg3 Mutations: Alteration of Ergosterol Biosynthesis

Fungi exposed to azoles suffer from ergosterol depletion and accumulation of toxic sterols, resulting in growth arrest (Kanafani and Perfect, 2008). Another less frequent mechanism of azole resistance is inactivation of the enzyme sterol Δ^5,6^-desaturase encoded by the gene ERG3, which is essential for ergosterol biosynthesis (Sanglard and Odds, 2002). Therefore, ERG3 protects yeast against toxic sterols; in contrast, deletions or mutations in ERG3 result in high levels of azole resistance once the production of toxic sterols is bypassed (Watson et al., 1989; Kelly et al., 1997). ERG3 mutants have been well studied in *C. albicans* and *C. dubliniensis* (Morio et al., 2012). Nonetheless, few of these mutations result in amino acid changes in Erg3 (Pinjon et al., 2003; Chau et al., 2005; Martel et al., 2010; Vale-Silva et al., 2012). Indeed, the exact mechanism by which a single substitution results in azole resistance needs to be investigated. Studies to date have demonstrated that cross-resistance between azoles and polyenes may occur because of ERG3 loss of function, which results in low ergosterol contents, protecting yeast against the toxic effects of AmB (Anderson et al., 2014; Sanglard, 2016). Some studies point to other mutations in genes of ergosterol biosynthesis suspected to be related to azole resistance, such as ERG6, ERG24, and ERG2 (Jensen-Pergakes et al., 1998; Jia et al., 2002; Vincent et al., 2013).

### Molecular Mechanisms of Resistance to Flucytosine

In relation to 5-FC, approximately 10% of *C. albicans* isolates present primary resistance, even in the absence of drug exposure (Arikan and Rex, 2005). Different studies have shown that resistance to 5-FC is related to its metabolism. In *C. glabrata* and *C. albicans*, mutation in cytosine deaminase confers primary resistance to 5-FC, and deficiencies in cytosine permease activity have also been associated with resistance in *Candida* species (Hope et al., 2004; Vandeputte et al., 2011). Cytosine permease is involved in the uptake of 5-FC, after which cytosine deaminase produces 5-fluorouracil using cytosine; thus, 5-FC resistance is associated with deficiency in enzymes related to uptake, transport and transformation of 5-FC that result in failure to metabolize to the active drug (Bondaryk et al., 2013). A *C. lusitaniae* mutant lacking the enzyme uracil phosphoribosyl transferase, encoded by the gene FUR1 (Papon et al., 2007), exhibits secondary resistance to 5-FC. The enzyme UPRT converts 5-fluorouracil to 5-fluorouridine monophosphate and inhibits thymidylate synthetase by disrupting DNA synthesis.

The molecular mechanism of the resistance of *Cryptococcus neoformans* to 5-FC is not well established but is often related to mutation in pyrimidine salvage enzymes, as occurs in *C. albicans* (Whelan, 1987).

Song et al. (2012) demonstrated that *C. neoformans* might possess different mechanism of resistance against 5-FC that are based on sensor histidine kinases. *C. neoformans* possesses a two-component system, Tco2 and Tco1, to regulate 5-FC response; deletion of *TCO2* leads to strong 5-FC resistance, and mutation in *TCO1* increases susceptibility. In addition, through transcriptomic analysis, it was found that 5-FC-regulated genes in *C. neoformans* differ from those of *S. cerevisiae.* As most of these genes are of unknown function in other fungi, *C*. *neoformans* appears to have a unique mechanism of resistance against 5-FC.

### Molecular Mechanisms of Resistance to Echinocandins

Mutations in the FKS1 gene lead to alterations in the conformation of the encoded enzyme, resulting in lower affinity between Fks1 and echinocandins and consequently resistance to these drugs (Gonçalves et al., 2016). Mutations in two hot spot regions of FKS1 are conserved in clinical isolates of *C. albicans*: the region between 641 and 648 (comprising a cytoplasmic domain/binding site of echinocandins) and 1345–1365 are hot spot 1 and hot spot 2 (HS2), respectively. These sites are responsible for the majority of mutations conferring resistance to echinocandins, including the most described: substitution of serine at position 645 (Balashov et al., 2006). Different studies have demonstrated alterations in FKS1 in other *Candida* species (Park et al., 2005; Desnos-Ollivier et al., 2008; Garcia-Effron et al., 2008, 2010), whereas mutations in Fks1 and its paralog Fks2 have been associated with resistance in *C. glabrata* (Garcia-Effron et al., 2009; Pham et al., 2014; Perlin, 2015).

*Candida parapsilosis* and *C. guilliermondii* show reduced susceptibility to echinocandins, most likely due to a natural polymorphism in the Fks1p hot spot region corresponding to mutations acquired in resistant isolates of other species: substitution of proline to alanine at positions 660 and 642 (Perlin, 2007; Garcia-Effron et al., 2008). Studies have shown that activation of cell wall recovery or compensatory pathways increases chitin production and that mutations that increase the chitin level result in reduced caspofungin susceptibility in *C. albicans* (Plaine et al., 2008).

Alterations in the Fks subunit have also been associated with echinocandin resistance in other fungi. A mutant with an amino acid substitution S678P in Fks1p resulted in resistance of *A. fumigatus* to echinocandins (Rocha et al., 2007). Alterations in FKS are also involved in *Fusarium* intrinsic resistance to echinocandin (Katiyar and Edlind, 2009).

*Cryptococcus neoformans* is intrinsically resistant to echinocandins without Fks alteration (Maligie and Selitrennikoff, 2005). Recently, Huang et al. (2016) investigated the molecular basis of *C. neoformans* resistance to echinocandins through a high-throughput genetic screen and found that the *CDC50* gene may be involved in echinocandin resistance. *CDC50* encodes the β-unit of membrane lipid flippase, which mediates the lipid trafficking pathway.

### Molecular Mechanisms of Resistance to Polyenes

Although the development of acquired resistance to AmB rarely occurs in *Candida*, there are some reports to date (Favel et al., 2003; da Matta et al., 2007). Resistance to AmB generated a common phenotype with alterations in the membrane lipid composition and consequently a change in fluidly and permeability. A greater number of cases of therapy failure of AmB have been associated with *C. lusitaniae* (Minari et al., 2001; Favel et al., 2003; Atkinson et al., 2008). The main alterations involved in polyene resistance are in enzymes participating in ergosterol biosynthesis. Defects in ERG2 and ERG3, encoding C-8 sterol isomerase (converting fecosterol to episterol with low affinity for AmB) and Δ^5,6^-desaturase, respectively, result in quantitative and qualitative modifications in the membrane sterol content, influencing the amount of ergosterol or its availability for the action of polyenes (Arikan and Rex, 2005; Sheikh et al., 2013). A defective ERG3 gene resulted in low ergosterol levels in the fungal membrane of fungal, conferring azole/polyene cross-resistance to *Candida* isolates. Another likely AmB-resistance mechanism is via enhanced activity of catalases, which reduce oxidative damage (Sokol-Anderson et al., 1986; Kanafani and Perfect, 2008).

Although the mechanisms of resistance to AmB in *Candida* spp. are well described, these mechanisms in non-*Candida* species remain unclear. Resistance to AmB in *C. neoformans* isolated from AIDS patients was linked to alterations in sterol delta 8-7 isomerase (Kelly et al., 1994). *Aspergillus* strains are commonly resistant to AmB, though this varies among species, without alteration in ergosterol content. One of the mechanisms proposed for *A. terreus* AmB resistance is blockage of the Ras signaling pathway by Hsp90 and Hsp70, inhibiting the formation of aqueous pores (Blum et al., 2013; Blatzer et al., 2015).

## Biofilm and Antifungal Resistance

The ability of many fungi to form biofilms is one of the reasons for antifungal drug resistance. Many medically important fungi are described as biofilm-forming organisms, such as *Candida* spp. (Chandra et al., 2005; Hinrichsen et al., 2008; Finkel and Mitchell, 2011; Pires et al., 2011), *Pneumocystis* spp. (Cushion et al., 2009), *Coccidioides* spp. (Davis et al., 2002), *Aspergillus* spp. (Mowat et al., 2009; Kaur and Singh, 2014), Zygomycetes (Singh et al., 2011), *Malassezia* spp. (Cannizzo et al., 2007; Figueredo et al., 2012), *Trichosporon* spp. (Di Bonaventura et al., 2006; Colombo et al., 2011), *Cryptococcus* (Walsh et al., 1986; Martinez and Casadevall, 2007), *Histoplasma capsulatum* (Pitangui et al., 2012), *Trichophyton* spp. (Costa-Orlandi et al., 2014) and *Paracoccidioides* spp. (Sardi et al., 2015).

Biofilms are highly structured and complex microbial communities embedded in a self-produced extracellular matrix (ECM) that attach to a wide range of surfaces and (Fanning and Mitchell, 2012). Several factors contribute to initial surface attachment, such as pH, temperature, osmolarity, flow of the surrounding biologic medium, host immune factors and even the presence of antimicrobial agents (Baillie and Douglas, 2000; Chandra et al., 2001; Ramage et al., 2008).

Fungal biofilm formation occurs through a sequential process including planktonic cell adhesion to an appropriate substratum, colonization, ECM production, biofilm maturation, and dispersion (Fanning and Mitchell, 2012). Despite these specific characteristics, all types of fungal biofilms have distinct properties from planktonic yeast cells and increase antifungal drug resistance up to 1000-fold (Ramage et al., 2001; Uppuluri et al., 2010). Indeed, several studies have shown the inefficacy of antifungal therapy against different fungal biofilms.

Multiple other biofilm-specific factors contribute simultaneously to the resistance of yeasts to antifungal drugs, including cell density, quorum sensing, eﬄux pump activity, persister cells, ECM presence, stress responses and overexpression of drug targets.

The cell density is an important factor that contributes to the antifungal resistance of biofilms. However, some studies show that this is not a biofilm-specific resistance mechanism because a similar trend was observed for planktonic cells. Perumal et al. (2007) studied the efficacy of different azoles, AmB and caspofungin on planktonic cells at densities similar to those found in biofilms. The susceptibility of dissociated biofilm cells was similar to that of planktonic cells at the same cell density, and this susceptibility decreased as the density of the cells increased (Perumal et al., 2007). This phenomenon was also demonstrated by Seneviratne et al. (2008), who noted the density-dependent susceptibility of planktonic or biofilm for ketoconazole and 5-FC. Lass-Florl et al. (2003) showed similar drug resistance results by increasing the inoculum sizes of *Aspergillus* species, supporting the idea that the physical density of the cells influences antifungal agent activity.

By providing microorganisms with the ability to communicate and coordinate population growth/morphology via the secretion of signaling molecules, cell density can be considered a key aspect of the quorum-sensing process (Ramage et al., 2009, 2012). QS for fungi was first described in *C. albicans* by Hornby et al. (2001), who identified farnesol as a molecule that inhibits the hyphal transitional stage of *C. albicans* and increases adhesion (Saville et al., 2003; Ramage et al., 2009). Exposing *C. albicans* to farnesol also resulted in alterations in gene expression involving hyphal developmental genes (*TUP1* and *CRK1*), cell surface hydrophobicity genes and those involved in drug resistance (*FCR1* and *PDR16*) (Cao et al., 2005; Enjalbert and Whiteway, 2005). In addition, farnesol induces apoptosis in both *A. nidulans* and *Fusarium graminearum* (Semighini et al., 2006, 2008). Tyrosol, the second QS molecule identified in *C. albicans*, promotes germ tube formation. According to Alem et al. (2006), tyrosol enhances the early phase of biofilm formation and may also inhibit farnesol activity, thereby controlling cell population morphology.

Khot et al. (2006) for the first time studied the mRNA levels of genes involved in ergosterol biosynthesis (ERG genes) and in β-1,6-glucan biosynthesis (SKN1 and KRE genes) in comparison between planktonic and biofilm-associated cells. The authors described the appearance of a unique transcript profile in a subpopulation of AmB-resistant blastospores with significant upregulation of ERG25, SKN1, and KRE1 and downregulation of ERG1. Mukherjee et al. (2003) reported that ergosterol levels were significantly decreased in intermediate and mature phases when compared to early-phase biofilms, suggesting that the biofilm resistance to azole might be explained by ergosterol alterations in biofilm membranes. Subsequent studies (Borecká-Melkusová et al., 2009; Nett et al., 2009; Nailis et al., 2010) compared the exposure of young and mature biofilms to fluconazole, concluding that both induce downregulation of genes encoding enzymes involved in ergosterol biosynthesis (Ca*ERG1*, Ca*ERG3*, Ca*ERG11*, and Ca*ERG25*). In addition, treatment of both young and mature biofilms with AmB predominantly resulted in overexpression of Ca*SKN1*, with only modest upregulation of Ca*KRE1* (Nailis et al., 2010).

Induction of ergosterol pathway genes has been described in different biofilms of *Candida* species. For example, incubation with fluconazole caused upregulation of Cd*ERG3* and Cd*ERG25* in *C. dubliniensis* (Borecká-Melkusová et al., 2009) and of genes involved in ergosterol biosynthesis in *C. parapsilosis*, resulting in antifungal resistance (Rossignol et al., 2009).

Moreover, drug eﬄux pumps also participate in drug resistance of biofilms. Ramage et al. (2002) investigated the role of eﬄux pumps, ABC and MFS transporters, of *C. albicans* biofilm. Expression of CDR genes predominated at the beginning of biofilm formation (24 h), whereas MDR1 was solely overexpressed after 24 h. Expression of CDR1, CDR2, and MDR1 showed that *C. albicans* was susceptible to azoles when grown planktonically but exhibiting resistance when grown in a biofilm. Several subsequent studies confirmed these results, suggesting that the expression of these genes is necessary for biofilm resistance (Mukherjee et al., 2003; Perumal et al., 2007). Gao et al. (2014) showed that fluconazole increased the expression of CDR1, CDR2 and MDR1 and that the combination with doxycycline downregulated the gene overexpression induced by FLZ.

Later, Nett et al. (2009) performed an *in vivo* study of *C. albicans* biofilm formation on implanted catheters and showed upregulation of genes during biofilm development: CDR2 at 12 h and MDR1 at both 12 and 24 h. Similar results were reported for other *Candida* species, including *C. glabrata*, in which the expression of *CDR1* and *CDR2* was found at early (6 h) and intermediate (15 h) biofilm stages, even though neither gene was upregulated at the mature phase (48 h) (Song et al., 2009). *C. tropicalis* showed increased expression of MDR1 after 24 h of biofilm formation (Bizerra et al., 2008). In *A. fumigatus*, the initial phase of biofilm formation was associated with increased activity of the eﬄux pump MDR and gene upregulation at 8 h when treated with voriconazole (Rajendran et al., 2011). Thus, all reports support the idea that eﬄux pump expression is a mechanism of biofilm resistance, especially in the early phase of biofilm growth until ECM production.

The ECM is considered one of the essential mechanisms of biofilms resistance, conferring enhanced antimicrobial resistance and protection from host immune responses (Sutherland, 2001; Davies, 2003). The ECM may act as an adsorbent, reducing the amount of antimicrobial available to interact with the biofilm, and the structure physically reduces the penetration of antimicrobial agents by walling off access to regions of the biofilm (Taraszkiewicz et al., 2013). The matrix is composed of a variety of proteins, nucleic acids, phospholipids, lipids, amyloid fibers, humid substances, and in some cases, surprising amounts of extracellular DNA (e-DNA) (Estrela et al., 2009; Dogsa et al., 2013). The ECM confers important characteristics to biofilm, such as providing for mechanical stability, an external digestive system, and intense cell interactions, including cell–cell communication and synergistic microconsortia, and serving as a nutrient, energy, and recycling source (Flemming and Wingender, 2010).

During biofilm formation, β-1,3 glucan is one of the principle carbohydrate components of the ECM (Ramage et al., 2012). It is responsible for sequestering azoles, echinocandins, pyrimidines, and polyenes, acting as a “drug sponge,” and confers resistance to *C. albicans* biofilms (Nett et al., 2010a,b). In addition, the ECM of non-*albicans Candida* strains also contains β-1,3 glucan, which contributes to azole resistance via specific binding (Mitchell et al., 2013). FKS1 was the first gene described as encoding β-1,3 glucan synthase of *C. albicans* (Nett et al., 2010a). Other genes essential for the *C. albicans* ECM are SMI1 and RLM1, which are involved in the protein kinase C cell-wall integrity pathway, controlling the cell wall glucan content in response to stress. Recently, Taff et al. (2012) showed that glucan transferases and exoglucanase are crucial for the accumulation and delivery of β-1,3 glucan to the matrix.

Biofilm presents persister cells, which are directly correlated with accumulating high concentrations of antimicrobial agents. In essence, a persister is a dormant cell that with little or no cell wall synthesis; drugs bind to their target molecules but are unable to promote cell death (Lewis, 2007). The simplest route to form a dormant persister cell might be through the overproduction of proteins that are toxic to the cell and inhibit growth (Lewis, 2010). Different from bacteria, these cells have only been detected in yeast biofilms and not in planktonic populations (LaFleur et al., 2006). Due to the ECM, the persisters present in the biofilm can withstand both antifungal treatment and the immune system (Lewis, 2007). Persisters may be mainly responsible for re-infection once when the concentration of antimicrobial decreases, and they can repopulate the biofilm (Lewis, 2001). Recently, a dose-dependent study involving AmB and chlorhexidine against *C. albicans* in planktonic and biofilm forms reported complete elimination of planktonic cells in both exponential and stationary stages. However, biphasic killing occurred in the mature biofilm, suggesting the presence of persisters. After AmB treatment, surviving *C. albicans* in the biofilm capable of producing a new biofilm with a new subpopulation of persisters, suggesting that yeast persisters are not mutants but phenotypic variants of the wild-type population (LaFleur et al., 2006). In addition, Sun et al. (2016) showed that *C. albicans* AMB-tolerant persisters were produced mainly during the adhesion phase and that the maintenance of these cells was dependent of surface adhesion. Further studies showed the presence of persister cells after the treatment of *C. krusei* and *C. parapsilosis* biofilms with AmB. However, the biofilm of *C. albicans* SC5314 under the same treatment condition did not reveal surviving cells, indicating a lack of persister cells (Al-Dhaheri and Douglas, 2008).

Based on the above, it is possible to affirm that biofilm confers antifungal resistance and that this occurs through various mechanisms. Accordingly, the discovery of new strategies to overcome these microbial communities has been deeply researched.

The development of new drug formulations is one of the main options of studies, including echinocandins and AmB lipid forms that are inhibit fungal biofilm both *in vitro* (Kuhn et al., 2002; Ramage et al., 2013) and *in vivo* (Mukherjee et al., 2009; Kucharikova et al., 2013).

The association of drugs is another example of a strategy against fungal biofilms. Some studies have reported the efficacy of a combination of AmB and aspirin (Zhou et al., 2012), FLZ and doxycycline (Gao et al., 2013), and caspofungin and diclofenac (Bink et al., 2012). The sensitization of *C. albicans* biofilms to different antifungals by the immunosuppressant drug cyclosporine A also resulted in enhanced biofilm inhibition (Shinde et al., 2012).

The presence of e-DNA, which is an important component of the ECM, has prompted some strategies using DNase to decrease biofilm biomass and to enhance the activity of antifungal agents (Martins et al., 2010). Lactonases and α-amylases are also used to control fungal biofilms (Taraszkiewicz et al., 2013). Moreover, an interesting therapeutic option is to block *persister* survival. Bink et al. (2011) discovered that superoxide dismutase (SOD) may be an inhibitor of *N,N*’-diethyldithiocarbamate (DDC) in *C. albicans* biofilms, reducing the miconazole-resistant *persister* fraction by 18-fold (Bink et al., 2011).

The application of antimicrobial photodynamic therapy has been investigated for anti-fungal biofilm properties with regard to the efficiency of inhibiting several microorganisms, with minimal damage to the host cell (Biel, 2010). This therapy involves the combination of photosensitizer (PS), light and molecular oxygen (de Melo et al., 2013). In addition, strategies involving nanoparticles (Allaker, 2010; Ramasamy et al., 2016), plant extracts (Wojnicz et al., 2012), and chitosan (Carlson et al., 2008) have been applied against microbial biofilm, with significant results. Further studies are necessary for identifying means to overcome fungal biofilm resistance.

## Drug Combinations

Based on the problems discussed above with regard to antifungal treatment, one of the options is the combination of drugs. Using more than one drug can increase efficacy due to the possibility of action on more than one target; in addition, toxicity is reduced because less of the drug is used (Chen et al., 2014). Drug combinations can lead to improved activity, such as synergist activity, or decrease antagonist action. Evaluation of the effect of a drug combination *in vitro* can be realized by the checkerboard method (Johnson et al., 2004).

Most studies on the *in vitro* and *in vivo* combination of azoles with AmB do not show synergistic activity, though previous treatment with azole can influence the action of polyenes. This can be explained by the fact that both drugs have the same target: ergosterol. However, because azoles inhibit ergosterol biosynthesis, less ergosterol is available for polyene to bind (Louie et al., 2001; Sanglard, 2002). Nonetheless, invasive mucormycosis has been successfully treat with an antifungal combination of AmB and posaconazole (Pagano et al., 2013).

There are numerous *in vitro* studies of antifungal combinations using triazole with echinocandin or triazole with AmB (Elefanti et al., 2013; Katragkou et al., 2014). Regardless, the clinical results of combinatory treatment remain unclear and are generally described for infections caused by fungi, which present difficulty in treatment, such as in aspergillosis and mucormycosis (Belanger et al., 2015). The combination of caspofungin and voriconazole showed *in vitro* synergistic activity against *Aspergillus* spp. (Walsh et al., 2008). Moreover, this combination showed efficacy in a mammalian model (Kirkpatrick et al., 2002). Treatment of patients with invasive aspergillosis with a combination of voriconazole and anidulafungin improved survival in comparison with treatment with voriconazole monotherapy (Marr et al., 2015). However, in another study, triazole and echinocandin combination showed the same effect as triazole alone (Raad et al., 2015).

The antifungal 5-FC in combination with AmB or with azole did not show synergistic activity for *Candida* sp. (Scheid et al., 2012). However, this was efficient for the treatment of cryptococcal meningitis. It should be noted that the lack of availability of 5-FC results in the use of less effective combinations in many countries (Perfect et al., 2010). Moreover, this compound is associated with rapid development of resistance (Vandeputte et al., 2011).

Interaction with non-antifungal agents has also been described as potentiating antifungal activity. Triclosan, a compound exhibiting antimicrobial activity, is widely used in soap, toothpaste, and other personal care products. This molecule showed *in vitro* synergistic activity with FLZ against *C. albicans* (Yu et al., 2011). Triclosan is also active against *C. neoformans* by activating the apoptosis pathway and also shows synergic activity with AmB and FLZ (Movahed et al., 2016). As ion homeostasis is a fundamental factor in the development of fungal disease, ion chelators have been used in the treatment of fungal infections. However, synergistic activity with antifungal drugs did not result in favorable responses *in vitro* (Lai et al., 2016). Treatment of cardio-vascular disorders has been realized with calcium channel blockers, which also demonstrate antifungal activity (Yu et al., 2014). Recent work shows that the calcium channel blockers amlodipine, nifedipine, benidipine, and flunarizine present synergistic activity with FLZ in *C. albicans* isolates resistant to FLZ via a mechanism not related to eﬄux pump inactivation (Liu S. et al., 2016).

Natural source molecules with antifungal activity are also described as having *in vitro* synergistic activity with traditional antifungal drugs, reducing the concentration of both substances (Soares et al., 2014; Sardi et al., 2016; Wang et al., 2016). Use of the natural substance beauvericin with traditional antifungals was able to inhibit eﬄux pumps and morphogenesis in FLZ-resistant *C. albicans* and potentiate the action of this azole (Shekhar-Guturja et al., 2016).

## New Antifungal Formulations and New Antifungal Drug Structure Modification

Two different strategies have been developed to increase the therapeutic index of antifungal agents: chemical modifications and/or elaboration of new formulations of antifungal agents to obtain less toxic derivatives (Sheng and Zhang, 2011).

Theoretical and experimental studies on the mechanism of action of AmB and its derivatives were performed by Borowski (2000). Two generations of derivatives were developed. First-generation compounds are modified at the carboxyl group, which improves selective toxicity based on disturbance of the hydrogen bond network in complex with sterols. Second-generation compounds have introduction of a bulky substituent, resulting in an appropriate steric hindrance effect that disturbs interaction with cholesterol but not with ergosterol, leading to improved selective toxicity (Borowski, 2000). Among second-generation derivatives, *N*-methyl-*N*-D-fructosyl AMB methyl ester (MFAME) is considered the most interesting compound because it is able to form water-soluble salts, has a broad antifungal spectrum, and lower toxicity than AMB toward animal cells in *in vitro* and *in vivo* experiments (Szlinder-Richert et al., 2001).

Despite the huge effort made to decrease fungal resistance and the toxicity of AmB, the development of rational chemical modification of known antifungal agents was insufficient to solve these problems. Thus, new delivery systems have been evaluated to reach this goal (Borowski, 2000; Sheng and Zhang, 2011).

Since 1990, nanostructured systems have been studied as carriers of antifungal agents. Clinically, the intravenous dosage form of AmB-deoxycholate has adverse effects, mainly nephrotoxicity. The synthesis of AmB analogs such as AmB esters or a preparation including an AmB lipid complex, AmB colloidal dispersion, liposomal AmB and intralipid AmB have been generated to improve the therapeutic index and lower toxicity (Gupta and Tomas, 2003; Vyas and Gupta, 2006; Voltan et al., 2016).

In addition, other delivery systems, such as carriers based on solid and nanostructure lipids, synthetic and natural polymers, inorganic and metal nanostructure lipids, dendrimers, silica, and carbon materials (magnetic nanoparticles), have been pursued. These delivery systems are able to improve bioavailability and reduce toxicity and present specificity for target tissues; however, there is an associated high cost of production (Vyas and Gupta, 2006; Sato et al., 2015; Voltan et al., 2016).

Recent work shows that the composition of nanoparticles used as delivery vehicles is fundamental for increasing antifungal activity. Ahmad et al. (2016) produced a conjugate system with AmB and metal nanoparticles that displayed synergistic antifungal activity due to the antimicrobial property of silver against *C. albicans* and *C. tropicalis*. Niemirowicz et al. (2016) reported synergistic activity for the combination of polyenes and magnetic nanoparticles. This bio-active nano-sized formulation exhibited enhanced efficiency against two clinical isolates of *Candida* species in planktonic and biofilm states.

For many years, the only available antifungal for invasive fungal infections was AmB, which has been incorporated into three lipid formulations. However, the imidazole class offers new treatment options because it is proven to be less toxic and in some cases more effective than AmB (Shalini et al., 2011). Although the imidazole class has been available for a decade, improvements in safety were necessary. Accordingly, this class was also subjected to rational chemical modifications. The triazole class was generated by the addition of a nitrogen atom to a cyclic ring. This modification provided a broad spectrum of activity as well as improved safety and pharmacokinetic profile (Allen et al., 2015). In this sense, the introduction of triazoles accelerated the pace of drug development.

New azoles have been developed to combat resistant pathogens to improve the tolerability and administration. Voriconazole is structurally similar to FLZ, with the exception of a fluoropyrimidine group in place of a triazole moiety, which leads to better bioavailability. Alternatively, posaconazole has a spectrum of antifungal activity comparable to that of voriconazole, but the molecular structure with a hydroxylated analog is similar to that of itraconazole. Currently, isavuconazole, ravuconazole, albaconazole, and efinaconazole are the four best studied agents in research aiming to identify an ideal antifungal with a wide spectrum and reliable pharmacokinetics, parenteral and oral dosage forms, and a favorable adverse effect profile (Shalini et al., 2011; Allen et al., 2015).

In addition, Moazeni et al. (2016) reported that the combination of solid lipid nanoparticles with FLZ was able to avoid recognition by eﬄux pump proteins, preventing extrusion when tested against FLZ-resistant *Candida* isolates. In another study, an aqueous nano-suspension of itraconazole for the treatment of bronchopulmonary aspergillosis solved pharmacokinetic problem of adequate concentration (Rundfeldt et al., 2013).

In addition to all azole agents, echinocandins are among the newest class of antifungal agents and act by inhibiting glucan synthesis in the fungal cell wall. Echinocandins are composed of a complex hexapeptide core with an *N*-terminus acylated by a long hydrophobic chain. Although echinocandins are fungicidal with good selectivity, they cannot be orally administered because of their complex lipopeptide structure. To address the limitation, several small molecule glucan synthesis inhibitors have been discovered. However, none is under clinical evaluation thus far (Sheng and Zhang, 2011; Liu N. et al., 2016).

## Antifungal Immunotherapy: Vaccines

Given their increasing frequency and unacceptably high morbidity and mortality rates, prevention of invasive fungal infections has become of vital importance (Spellberg, 2011; Medici and Del Poeta, 2015). Vaccination of high-risk groups is a particularly promising strategy to prevent invasive fungal infections because easily identifiable risk factors are clearly defined for many such infections (Perlroth et al., 2007; Spellberg, 2011).

Advances in our understanding of the host defense and pathogenic mechanisms underlying fungal infections has supported the development of effective vaccines to combat these diseases. Accordingly, researchers have dedicated studies to developing robust, durable and safe fungal vaccines, especially those that may be useful for endemic infections or in chronic or superimposed infections in intensive care patients (Santos and Levitz, 2014; Shahid, 2016).

Although very few clinical trials have been performed in humans, a growing number of antifungal vaccine candidates are being evaluated in pre-clinical studies. This may be due to the renewed interest in the potential use of vaccines, replacing or associated with chemotherapy, to reduce antifungal drug use and consequently limit drug resistance and toxicity.

For most active vaccines studied against invasive fungal infections, the key to protection has been the induction of cell-mediated, pro-inflammatory, Th1 or Th17 responses, which improve phagocytic killing of the fungus. It is also clear that antigens targeted for vaccination need not be restricted to virulence factors, markedly increasing the antigen repertoire available for testing. Additionally, the concept of niche vaccination of acutely at-risk patients or patients in restricted geographical areas is a new idea that opens doors in vaccinology (Spellberg, 2011).

The greatest advances in fungal vaccines are with regard to studies of invasive candidiasis, and two promising vaccines are presently in the clinical trial phase. The first, containing the rAls3p-N antigen, is in Phase IIa and prevents fungal adhesion and invasion in immunized hosts. Protection with this vaccine has been found to largely be mediated by T cells via neutrophil recruitment and a specific antibody in vaccinated hosts (Edwards, 2012; Shahid, 2016).

The second candidal vaccine in clinical trial phase is a virosome-based vaccine containing a Sap2 antigen/truncated recombinant Sap2 antigen. The truncated form is stable, immunogenic and harmless. The Sap2 vaccine administered intra-muscularly or intra-vaginally induces systemic and 100% mucosal protective immunity (Vecchiarelli et al., 2012; Shahid, 2016).

Another important molecule that has been studied in candidiasis vaccination is heat shock protein 90 (Hsp90-CA). Raska et al. (2005) tested an Hsp90-CA DNA vaccine and found that vaccinated mice exhibited survival times prolonged by 64% compared with the untreated control. In addition, vaccination with the recombinant protein r-hsp90-CA significantly increased survival compared to the control groups.

A monoclonal antibody that binds to the immunodominant epitope of *C. albicans* Hsp90p was tested pre-clinically. A synergistic action with AMB against FLZ-sensitive and -resistant *C. albicans* strains was demonstrated in *in vivo* and *in vitro* experiments. The same antibody was used in a multicenter double-blind placebo-controlled trial of patients with invasive candidiasis, and complete mycological resolution reached 84% in the combination therapy group compared to 48% in the AMB monotherapy group. Moreover, clearance of infections in the combination therapy group was twice that of AMB therapy alone, resulting in 4 and 18% mortality, respectively (Matthews et al., 2003; Bugli et al., 2013; Wang et al., 2015).

In a different approach, *S. cerevisiae* cells were genetically engineered to display Enolase 1 (Eno1p) antigens of *C. albicans* on their surface. Oral administration of these cells could elicit an immune response and aid the survival of mice challenged with *C. albicans* (Vecchiarelli et al., 2012; Shibasaki et al., 2013; Shahid, 2016). Interestingly, by sharing some elements with *Aspergillus* species, *Candida* enolase also exerts a protective function against aspergillosis.

Studies on vaccines protective against other fungi are still in the early pre-clinical phase (Fernandes et al., 2011; Hsieh et al., 2011; Edwards, 2012; Hole and Wormley, 2012; Shahid, 2016).

For protection against aspergillosis, studies have shown that the type 1, cell-mediated immune response, which is efficient in protecting against this disease, can be induced by recombinant protein antigens from *Aspergillus*, as observed by Bozza et al. (2002). Administration of recombinant allergen Asp 16 f in conjunction with CpG oligonucleotides improved the survival of mice infected by inhaled *A. fumigatus*. Vaccination with crude antigen preparations from *A. fumigatus* was also tested, and it was observed that such vaccination improved the survival of mice infected by inhaled and intravenously administered fungi, even in those that were subsequently immunocompromised (Cenci et al., 2000; Ito and Lyons, 2002).

In the case of *Cryptococcus*, Devi et al. (1991) proposed the use of an anti-phagocytic antigen from the capsule of *C. neoformans*, glucuronoxylomannan (GMX), as a vaccine to elicit antibody-mediated protection. In a different approach, Wozniak et al. (2011) administered T-cell depleted mice, mimicking a T-cell-deficient host, with an engineered strain of *C. neoformans* that could express IFN-g. After the immunization, a secondary pulmonary infection using a pathogenic strain was produced, and the mice were protected against the infection. The results demonstrate that it is possible to generate a protective immune response to *Cryptococcus* even after becoming immunocompromised, such as in cases of HIV.

Recently, Rella et al. (2015) proposed the use of a live attenuated mutant strain lacking sterol glucosidase enzyme (Δsgl1) as a vaccine. In this study, immunization of mice with Δsgl1 cells led to strong protection against challenge with *C. neoformans* and *C. gattii* and could also elicit protective immunity in mice deficient for T CD4+ cells, a frequent condition in cryptococcosis patients.

Another possible therapeutic agent in the prophylaxis of *Cryptococcus* is glycosphingolipid glucosylceramide (GlcCer), a *C. neoformans* virulence factor. In a recently study, Mor et al. (2016) demonstrated that administration of GlcCer prior to infection with *C. neoformans* in a murine model prevented dissemination of the fungi from the lungs to the brain and led to 60% mouse survival while preventing side effects such as hepatic injury, a common and great problem in the use of the antifungal therapy currently available.

A peptide derived from the gp43 adhesin of *P. brasiliensis*, named p10, protects mice from paracoccidioidomycosis (PCM), and combination of immunization with this peptide and different antifungal drugs showed an additive protective effect. The finding suggests that this is an important molecule to be tested in clinical trials against PCM (Taborda et al., 1998; Marques et al., 2006; Marques et al., 2008; Rittner et al., 2012).

To date, few vaccines have reached the clinical stage. However, the importance of mycosis to public health around the world indicates that efforts should be given to the discovery of new molecules and compounds that can be used in the prophylaxis and treatment of these diseases. The challenges are numerous, but the efforts offer hope for controlling these diseases that tend to increase in incidence over time.

## Alternative Animal Models to Study Fungal Virulence and Antifungal Drugs

Classically, mammalian models are considered the gold standard for drug discovery, virulence and immune response studies. However, in 1959, the concept of the “alternative animal model” was described by Burch and Russell with “the three R’s: Refinement, Reduction, and Replacement” (Russell and Burch, 1959). In **Table 1** are described the three R concept and the actions required for its application. In recent decades, the use of alternative animals, also called “mini hosts” or “non-conventional animal models” have been encouraging for *in vivo* testing. Amoeba, insects, nematodes, fish and chicken embryos are used for the following reasons: (1) because the neural systems of these animals are poorly developed, they are almost painless; (2) a large number of animals can be used for each experiment; (3) maintenance is cheaper than for traditional animals; (4) there is a good correlation between alternative animals and mammalian animals (Trevijano-Contador and Zaragoza, 2014).

**Table 1:** Burch and Russell: The three R’s concept and the action required for its application.

3 R rule	Action required
Reduction	Careful experimental design
	Consult the literature
	Organize a pilot experiment before using a bigger amount of animals
	Realize an appropriate statistical test
	Get the great amount of data from each animal
Refinement	Eliminate the pain and search for better alternatives for animal welfare
	Be trained before execute the procedures
	Use adequate doses of analgesics and anesthetic for the painful procedures
	Perform post chirurgical procedures like (e.g., thermoregulation)
Replacement	use *in silico* methods (e.g., computer software)
	use *in vitro* methods (e.g., cell culture and cell tissue)

Chicken embryos have been used since 1971 for fungal virulence studies (Partridge et al., 1971), and many studies have been published using this model to evaluate virulence of different fungi such as *Candida* spp., *Rhizopus*, and *A. fumigatus* (Jacobsen et al., 2010, 2011; Kaerger et al., 2015). Moreover, with this model, it is possible to evaluate the role of genes using mutants and to study the immune response of the host during infection (Jacobsen et al., 2010). Despite the importance of this model to date, experiments of antifungal efficacy have not been validated. Although chicken embryos are not consider worldwide to be living organisms and the ethical issues for this type of experimentation are less complicated, it is important to highlight that the euthanasia performed after experiments should be administered properly using anesthesia (Aleksandrowicz and Herr, 2015).

Another non-conventional model widely used for *in vivo* testing is zebrafish embryos, larvae, and adults. The main advantage is that it is possible to analyze different biological processes due to the presence of organs and systems, providing complete raw data analysis for different fields of science (MacRae and Peterson, 2015). This model was useful for studying of the *C. albicans* virulence (Chao et al., 2010; Brothers et al., 2011; Chen et al., 2015) and C. *neoformans* pathogenesis (Tenor et al., 2015). Similar to chicken embryos, there is no description thus far of the evaluation of antifungal efficacy using zebrafish. However, toxicity assays and teratogenic testing have provided important information. It is essential to highlight that after 120 h post-fertilization (hpf), ethical regulations are required for zebrafish experiments (Strähle et al., 2012).

Insects can also be used as an alternative animal model. The fruit fly *Drosophila melanogaster* is described as a model for investigating the virulence of human pathogens, due to similarities between its immune system and that of mammals, and for verifying the efficacy of novel antifungal compounds. Infection can be achieved by injection, rolling contact or ingestion (Lionakis and Kontoyiannis, 2012). Different *Candida* species have been evaluated using this model; the virulence of *C. parapsilosis* was found to be lower than that of *C. albicans*, similar to observations in humans; FLZ also increased the survival of susceptible strains (Chamilos et al., 2006). This model is also suitable for virulence evaluation of *C. neoformans* mutants (Apidianakis et al., 2004). The virulence of clinical and environmental isolates of *A. flavus* have been evaluated, as well as different mating types (Ramírez-Camejo et al., 2014). In *Drosophila melanogaster*, antifungal treatment is realized by ingestion, mixed with food. This model showed a correlation with mammalian models with regard to synergism of tacrolimus with posaconazole for the treatment of *Rhizopus oryzae* (Lewis et al., 2013). *Fusarium moniliforme* and *Scedosporium apiospermum* are able to infect *D. melanogaster*, and ingestion of food containing voriconazole increased the survival of infected flies (Lamaris et al., 2007).

*Galleria mellonella* has been described in different fields of science for numerous purposes, and the number of studies is increasing every year. Concerning virulence fungal assays, studies have been reported using *G. mellonella* as an infection model for different *Candida* species (Scorzoni et al., 2013; Frenkel et al., 2016; Moralez et al., 2016), *Cryptococcus* spp. (Benaducci et al., 2016), *Sporothrix schenckii* (Clavijo-Giraldo et al., 2016), *Paracoccidioides* spp. (Thomaz et al., 2013; Scorzoni et al., 2015), *Histoplasma capsulatum* (Thomaz et al., 2013), and *Fusarium* sp. (Coleman et al., 2011) and also for biofilm formation and behavior studies (Fuchs et al., 2010a; Benaducci et al., 2016).

The advances of this model are due to the ease of manipulation, low cost, and large range of larval incubation temperatures (25–37°C) with the possibility of mimicking the human body temperature. However, one disadvantage is that the genome is not fully sequenced, resulting in a lack of mutant strains to study host responses. Most of the work related to infection and treatment of *G. mellonella* has been via injection through one of the prolegs (Fuchs et al., 2010b). The benefit of this is that the inoculum and the treatment are controlled. *G. mellonella* is useful for *in vivo* treatment with conventional antifungal drugs (Coleman et al., 2011; de Lacorte Singulani et al., 2016), for assessing antifungal synergistic activity (Gu et al., 2016; Sangalli-Leite et al., 2016) and for evaluating the *in vivo* efficacy of new drug candidates (Browne et al., 2014).

The nematode *Caenorhabditis elegans* is been used for decades in different fields of biology. This organism has a short life cycle, produces a large number of progeny, and is transparent; moreover, the genome is fully sequenced with the possibility of studying host pathways through RNA interference (RNAi) techniques or constructing transgenic strains. *C. elegans* is maintained on agar medium and fed non-pathogenic *E. coli* (Muhammed et al., 2016). *C. elegans* is susceptible to infection by human pathogenic yeasts and filamentous fungi (Johnson et al., 2009; Pukkila-Worley et al., 2009; Desalermos et al., 2015). For this reason, this organism can be used for high-throughput screening of antifungal drugs candidates. The *C. elegans* model of infection is well described in antifungal drug discovery. A study with 1,266 compounds identified 15 anti-candidal substances that prolonged the survival of infected larvae and inhibited *in vivo* filamentation of the yeast (Breger et al., 2007). In another screen with 3,228 candidate substances, nine compounds with potential antifungal activity were identified (Okoli et al., 2009). Synergistic antifungal activity of tyrocidines and caspofungin was successfully evaluated *in vitro* and *in vivo* using *C. elegans* (Troskie et al., 2014). Moreover*, C. elegans* is a suitable model for studying the immune response because it is able to produce different antimicrobial peptides that are regulated upon infection; these peptides can also serve as new antimicrobial candidates (Ewbank and Zugasti, 2011).

Although different types of animal models are discussed here, it is important to evaluate which is the best system for each study. Regardless, although alternative models have benefits, mammalian models are still considered the gold standard, and many additional studies are necessary before the complete substitution of mammalian models for alternative animals. At present, alternative animals are suitable for screening prior to further studies in mammals.

## Conclusion

Despite the increasing number of reports regarding advances in antifungal therapy, the number of cases of infection and antifungal resistance are still alarmingly high, and control of antifungal disease is far from being achieved. Important new advances have been made in the discovery of antimicrobial fungal targets; however, many years are necessary from discovery to clinical use. Because of this, improving existing molecules and developing new formulations and alternative therapy for prevention and treatment are important for treating fungal infections and increasing treatment options and quality of life.

## Author Contributions

All the authors contributed selecting the topics, reviewing the literature, selecting important and atual informations. Moreover the authors read and approved the final manuscript.

## Conflict of Interest Statement

The authors declare that the research was conducted in the absence of any commercial or financial relationships that could be construed as a potential conflict of interest.
